# Public Health Investment in Team Care: Increasing Access to Clinical Preventive Services in Los Angeles County

**DOI:** 10.3389/fpubh.2018.00017

**Published:** 2018-02-08

**Authors:** Tony Kuo, Noel C. Barragan, Heather Readhead

**Affiliations:** ^1^Division of Chronic Disease and Injury Prevention, Los Angeles County Department of Public Health, Los Angeles, CA, United States; ^2^Department of Epidemiology, UCLA Fielding School of Public Health, Los Angeles, CA, United States; ^3^Department of Family Medicine, David Geffen School of Medicine at UCLA, Los Angeles, CA, United States; ^4^Department of Social Welfare, UCLA Luskin School of Public Affairs, Los Angeles, CA, United States

**Keywords:** clinical preventive services, access to care, team care, community partnerships, health policy

## Abstract

As part of federal and local efforts to increase access to high quality, clinical preventive services (CPS) in underserved populations, the Los Angeles County Department of Public Health (DPH) partnered with six local health system and community organization partners to promote the use of team care for CPS delivery. Although these partners were at different stages of organizational capacity, post-program review suggests that each organization advanced team care in their clinical or community environments, potentially affecting >250,000 client visits per year. Despite existing infrastructure and DPH’s funding support of CPS integration, partner efforts faced several challenges. They included lack of sustainable funding for prevention services; limited access to community resources that support disease prevention; and difficulties in changing health-care provider behavior. Although team care can serve as a catalyst or vehicle for delivering CPS, downstream sustainability of this model of practice requires further state and national policy changes that prioritize prevention. Public health is well positioned to facilitate these policy discussions and to assist health system and community organizations in strengthening CPS integration.

## Introduction

Multidisciplinary team care approaches, including the Patient-Centered Medical Home (PCMH) model, are broadly accepted sets of practice principles that can be used to improve health services delivery (1–5). Although generally accepted by clinicians and health system administrators alike, meaningful implementation of team care has been proven to be challenging, even in highly motivated outpatient settings (5). For example, team care’s focus on organizational development and coordination of health and community services often requires significant investment of time and other resources (5, 6). In addition, workflow redesigns are often difficult to implement because of competing, reimbursable priorities.

In 2011, the Centers for Disease Control and Prevention established the Community Transformation Grants (CTG) as part of the Patient Protection and Affordable Care Act of 2010 (ACA), Prevention and Public Health Fund (7, 8). The initiative had five key components: (1) tobacco free living; (2) active living and healthy eating; (3) high impact clinical and other preventive services; (4) social and emotional wellness; and (5) healthy and safe physical environment. In this article, we focus our attention on the Los Angeles County (LAC) Department of Public Health’s (DPH’s) effort to implement the third CTG component. We provide a perspective on the use of team care to increase patient access to high quality, clinical preventive services (CPS).

Beginning in 2011, DPH invested in variations of team care programming through partnerships with health systems and community organizations that served priority populations in LAC. In spite of growing interest in team care, there remains limited literature on how local public health agencies can help establish, enhance, and/or sustain this model of practice in a range of community and clinical settings (6). The present discussion highlights DPH’s experiences and lessons learned from six partnership efforts that took place during 2011–2014.

## Program Model and Implementation

### Context

DPH serves the most populous jurisdiction in the United States, spanning more than 4,000 square miles of urban, suburban, and rural settings (9). Home to more than 10 million people, LAC is characterized by tremendous diversity. There are over 200 different languages spoken by individuals representing more than 140 cultures and a vast number of multiethnic communities (10). The county is also home to a complex policymaking environment, including a large unincorporated area governed by the *County of Los Angeles* Board of Supervisors and 88 incorporated cities governed by mayors and city councils.

In spite of ongoing efforts, the county has faced numerous challenges in addressing health disparities, which stem from lack of access to coordinated clinical and other preventive services. For example, in 2015, 23.6% of LAC adults reported difficulty in accessing medical care, with the underserved regions having the greatest level of difficulty [Metro Service Planning Area (SPA)—28.6% and South SPA—32.5%] (11). In another example, only 44.8% of LAC adults aged 50 years or older received recommended colorectal cancer screening, lower than the target (70.5%) recommended by *Healthy People 2020* (12).

Evidence suggests that teamwork, care coordination, and continuity are key elements for improving the quality and efficiency of primary care delivery, including CPS delivery (5, 13, 14). In recent years, several local health-care organizations [e.g., LAC Department of Health Services (DHS)] have moved toward a team care approach for delivery of these services. However, team care is not widely understood or accepted by patients. In a 2014 survey commissioned by DPH, only 31% of respondents indicated high interest (*very interested* or *extremely interested*) in being treated at a clinic that uses team care (see Table SA in Supplementary Material). Even fewer expressed interest in the use of health navigators or health coaches (23%), both of which are common components of team care.

### Program Model

Team care, in all of its configurations, provides comprehensive care for a patient’s physical and mental health needs through a team of health care and social services providers that could include physicians, nurses, pharmacists, nutritionists, social workers, health educators, medical assistants, and community health workers (1). The approach is patient focused and encourages service providers to involve the patient as an informed partner in his/her care (4). Emphasis is placed on coordinating access to services across the broader health care system, making the experience more meaningful and efficient. Effective implementation of team care typically requires adoption and integration at multiple levels of the health care system, requiring support tools such as disease registries, electronic health records, decision support, provider reminder systems, and trainings to work in a team environment (4, 13). In LAC, health organizations that serve priority populations are at initial stages of this system-level transformation. CTG provided an unprecedented opportunity to explore and foster the integration of team care to increase patient access to high impact CPS.

**Figure 1:**
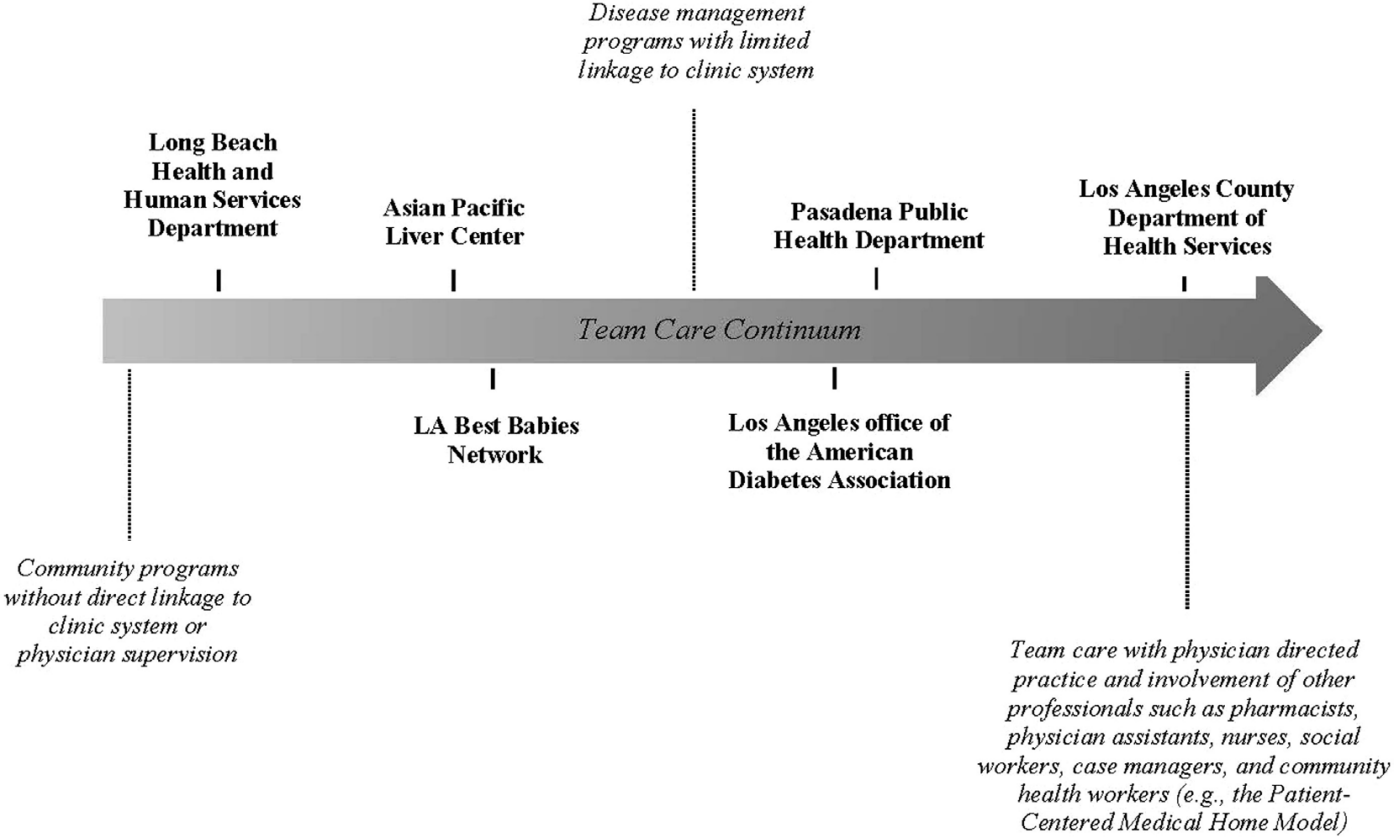
Team care variation among health system and community organization partners working to improve access to clinical preventive services in Los Angeles County.

During 2011–2014, a system change strategy utilizing team care principles was adapted by six local health system and community organization partners. Each entity was tasked with addressing gaps in their standard delivery of the “ABC’s” of CPS, namely, aspirin use in appropriate age and patient groups; blood pressure control; cholesterol management; and tobacco cessation utilizing the standard protocol “Ask, Advise, Refer” (8). Selection of the six partners was informed by a number of regional and operational factors, including (a) the requirements of the funding source; (b) the readiness of the system or program to engage in a team care model (e.g., existing plans to convert to PCMH, having an established billing infrastructure for group work, and a workforce trained or being trained to function in a team environment); (c) jurisdictional considerations (e.g., inclusion of the other two local health departments in LAC—Long Beach and Pasadena); (d) opportunities to leverage existing relationships in the local health care market; and (e) the needs of priority populations (e.g., lower utilization of CPS among Asians). These factors formed the foundation of DPH’s partnership approach to scale and spread team care in LAC (see Figure 1 for a snapshot of team care variation among the six partners).

### Program Partners and Their Use of Team Care

The Long Beach Department of Health and Human Services offers a free 6-week Diabetes Prevention and Management Program designed to teach adults with prediabetes and diabetes and their family members on ways to prevent and manage diabetes. The goals of the program are to increase knowledge about the management and prevention of the disease, to increase self-management behaviors and utilization of appropriate health services, and to ultimately improve the health outcomes of adults living with diabetes. Under this partnership, steps were taken to enhance CPS delivery by including additional screening, education, and referrals for tobacco use, alcohol use, and depression.

Through a community-based network, the Asian Pacific Liver Center (APLC) provides culturally tailored programming to a high-risk Asian community in Los Angeles. APLC provides comprehensive screening, vaccination, and preventive education for those at risk of developing viral hepatitis. Through the collaboration with DPH, APLC integrated blood pressure screening and tobacco screening into their outreach protocols and implementation, and expanded planning to include other potential screenings and referral support.

The LA Best Babies Network (LABBN) is dedicated to achieving healthy pregnancies and births in LAC by providing the infrastructure, programs, advocacy, and support to increase the capacity of community partners to succeed in these efforts. As part of the collaboration with DPH, the Network served as the Team Leader for the LA County Healthy Weight Collaborative, a national pilot for the Breakthrough Series Learning Collaborative™. The Network team brought together organizations, including several federally qualified health centers (FQHCs), that share a commitment to improving preventive and treatment services, specifically services that reduce cardiovascular disease and diabetes risk. LABBN offered intensive education, tools, and technical assistance for implementing clinical quality improvement projects utilizing multidisciplinary care teams.

The American Diabetes Association’s (ADA) Education Recognition Program endorses the National Standards for Diabetes Self-Management Education and Support and is one of the only two Centers for Medicare and Medicaid Services certifying bodies for diabetes self-management training. ADA recognition ensures that clinics are offering high quality, standardized care to their clients and provides clinics an opportunity to bill for diabetes education and related services.

Achieving ADA recognition can be time and resource intensive, and thus clinics with limited resources are often not able to independently seek recognition. As part of their DPH supported effort, the ADA was asked to actively recruit low-income clinic/service providers and facilitate their achieving ADA recognition. ADA provided these organizations with extensive technical assistance to develop protocols and infrastructure needed to achieve the required clinical care standards; application submission support; and funding to cover the $1,100 application fee. Additionally, ADA was asked to strengthen their Community of Practice, which provided local ADA recognized programs with an opportunity to share best practices and lessons learned. Some of these best practices included strategies for delivering the ABC’s of CPS in a clinic or health education setting.

The Pasadena Public Health Department (PPHD) serves the city of Pasadena in the county of Los Angeles. The department administers the Prevention, Adherence, Collaboration, Education (PACE) program for people over the age of 55 with type II diabetes. Patients are enrolled through the partnering FQHC, the Community Health Alliance of Pasadena (CHAP). Utilizing evidence-based concepts from the Chronic Care Model, the PACE program aims to reduce cardiovascular morbidity and mortality of patients with type II diabetes, improve quality of care in community clinics, and increase self-management of diabetes.

Prevention, Adherence, Collaboration, Education care management includes one-on-one patient diabetes education, assessment for appropriate referrals (retinal screening, ophthalmology, podiatry, clinical pharmacy, health education classes, physical activity classes, social services, and behavioral health), follow-up on lab testing and referral status, assistance with navigating the health care system, appointment reminders, chart review for medication adherence, and counseling on behaviors relating to nutrition and physical activity. PACE also provides outreach education to providers, which includes information on the A-L-L drug regimen (using aspirin, lisinopril or other anti-hypertensive medication, and a lipid-lowering statin), healthy eating, and physical activity to prevent diabetes and hypertension complications.

In collaboration with DPH, PPHD codified additional protocols that were incorporated into the PACE program including screening for tobacco use and referral to tobacco cessation resources (“Ask, Advise, Refer”) for all patients with type II diabetes seen by a primary care provider in CHAP clinics; proactive panel management of both diabetic and prediabetic patients through chart review; and referrals to a care manager, nutritionist, and nutrition and physical activity classes.

The LAC DHS is the second largest public-sector health system in the nation, serving more than 600,000 unique clients every year. DHS comprises 4 major hospitals and 19 comprehensive health centers. In this team care project, DHS was given the opportunity to accelerate the implementation of its plan to transform siloed personal health care delivery to the use of a PCMH-inspired model of care. This transformation process included structural changes made to the disease registry and electronic health records system, utilization of *expected practice* guidelines, and promotion of team care delivery protocols for use by DHS clinic providers and staff. Some of the work included:

(a) Hypertension/prehypertension and obesity identification and referrals embedded as part of the clinic disease registry/electronic health records system. As part of the protocol, referrals to further services and/or linkages to community resources that address lifestyle modification became a common feature in the clinical environment.
(b) Tobacco use screening, implementing the “Ask, Advise, Refer” brief intervention with follow-up on the referrals to the quit line and related treatment services.

This team care effort was first piloted at 6 sites, and then later expanded to 95+ DHS-operated and -affiliated facilities over a 12- to 18-month period.

## Lessons from the Field and Implications for Policy Development and Practice

Although DPH was successful in engaging and supporting six major health system and community organizations to improve access to CPS in the county (potentially impacting upwards of 250,000 clinic visits per year), the actual population reached and institutionalization of these services varied and was difficult to fully monitor. Throughout the process, DPH documented several barriers and facilitators to team care (see Table 1).

**Table 1:** Barriers and facilitators of team care for clinical preventive services (CPS) delivery in Los Angeles County (LAC), 2011–2014.

Partnering health system or community organization	Barriers	Facilitators
Long Beach Department of Health and Human Services	Data collection database tools required technical expertise to incorporate CPS program expansion, proving to be a significant barrier to outcome evaluation Lack of co-location/proximity to health promotion resources Lack of long-term funding	Leveraging of existing program infrastructure and other resources Program leadership support American Diabetes Association (ADA) recognition allowed for potential long-term sustainability *via* traditional medical billing

Asian Pacific Liver Center at St. Vincent Medical Center	Lack of co-location/proximity to health promotion resources Lack of long-term funding Categorical funding proved to be challenging when developing a program focused on both chronic and infectious conditions. Funding restrictions limited the potential synergy of expanding the scope of the program, which was initially focused on Hepatitis B	Leveraging of existing program infrastructure and other resources Cultural tailoring and long-standing community relationships proved essential to the program achieving good reach St. Vincent Medical Center (as parental organization) was committed to the community-based approach

LA Best Babies Network, Dignity Health	The rigor of program made it challenging to implement it in the clinical setting which often is not prepared to quickly make significant changes Lack of co-location/proximity to health promotion resources Not enough attention placed on sustainability planning (lack of long-term funding)	Leveraging of existing program infrastructure and other resources Commitment of organizational leadership to implement changes Physician champion Well-organized onsite and remote technical assistance with ready-to-use clinical quality improvement tools Team commitment to implementation including protected meeting/project time Financial incentives for partnering federally qualified health centers (FQHCs) based on quality metrics

American Diabetes Association (Recognition Program)	Voluntary organization infrastructure, not necessarily linked to clinical settings Time it takes for the recognition program to offer technical support and proactive recruitment Lack of co-location/proximity to health promotion resources Lack of long-term funding	Leveraging of existing program infrastructure and other resources Extensive technical support available to implement changes Proactive recruitment strategies Recognition program provides opportunity to establish capacity and standards that can lead to reimbursement of prevention services Financial incentives for partnering FQHCs and other managed care providers based on quality metrics

Pasadena Public Health Department	Coordination with local FQHCs and other clinical settings required new systems and infrastructure to support Rigorous interventions are challenging to scale up for larger population reach due to limited capacity Lack of long-term funding	Leveraging of existing program infrastructure and other resources Commitment of organizational leadership to implement changes Physician champion Other existing sources of financial support ADA recognition allowed for potential long-term sustainability *via* traditional medical billing Shared electronic health records between FQHCs and outside program allowed for better patient care coordination and program evaluation Financial incentives for FQHC partners based on quality metrics and Patient-Centered Medical Home (PCMH) certification status

LAC Department of Health Services	Need for provider training on team care and meaningful use of disease registry and electronic health record system Lack of co-location/proximity to health promotion resources Lack of community resource inventory Lack of long-term funding	Leveraging of existing program infrastructure and other resources Commitment of organizational leadership to implement changes Physician champion Affordable Care Act requirements and reimbursement incentive related to CPS Anticipated financial incentives for managed care providers based on quality metrics and PCMH certification status

### Key Barriers

Health system and community organization partners experienced several barriers that were common across settings. Lack of co-location with, or proximity to, community resources that could benefit patients and the need for easy access to information about existing resources were common challenges expressed by the partners (*n* = 5 organizations, out of 6). Reliance on short-term grant funding and lack of earmarked financial support from parent organizations also impeded consistent delivery and integration of CPS (*n* = 6). APLC, for example, saw its community-based CPS program sunset once DPH funding support was not available. This was in spite of the blood pressure screening and clinic referral program’s success, reaching nearly 1,500 people in just a little over 6 months (15).

### Key Facilitators

Despite the variation in team care infrastructure and implementation (Figure 1), there were a number of shared facilitators that supported greater integration and broader reach of the CPS strategy. These included having a firm commitment from organizational leadership to implement the system changes (*n* = 3); features of the ACA that promoted reimbursement incentives for prevention services (*n* = 4); having physician champions (*n* = 3); and building CPS programming in alignment with the goals of other chronic disease management programs (*n* = 6).

Additionally, DPH’s effort to scale team care created new opportunities for the six health system and community organization partners to network and collaborate, benefiting from each other’s work. For example, out of the five organizations that actively engaged in the ADA recognition process, two of them were the Long Beach and Pasadena health departments.

## Conclusion

Despite existing infrastructure and DPH’s funding support of CPS integration, partner efforts continued to face key challenges. These included lack of sustainable funding for prevention services; limited access to community resources that support disease prevention; lack of co-location and proximity to health promotion resources; and difficulties in changing health care provider behavior (4, 5). The experiences described in this narrative suggest that further research is needed to clarify effective pathways for translating team care theory to meaningful practice. A better understanding of how team care implementation varied across these diverse settings could provide valuable insights and suggestions for organizations looking to creatively support CPS integration within existing program infrastructure.

Although team care can serve as a catalyst or vehicle for delivering CPS, downstream sustainability of this model of practice requires further state and national policy changes that prioritize prevention (1, 2, 5). Public health is well positioned to facilitate these policy discussions and to assist health system and community organizations in strengthening CPS integration. In the present narrative, DPH’s experiences are shared to provide information that can be used by other jurisdictions and decision-makers to develop their own roadmaps for achieving health equity and better delivery of CPS.

## Author Contributions

TK and NB conceptualized the framework for the writing of this article. TK and NB also gathered and synthesized the information contained in the tables, figures, and text. NB compiled and summarized the quantitative data. HR provided programmatic input on the content presented. All authors reviewed and assisted with the writing of the article. All authors have reviewed and approved this submission.

## Conflict of Interest Statement

The authors declare that the research was conducted in the absence of any commercial or financial relationships that could be construed as a potential conflict of interest.
