# Supplementary material for: Public Health Investment in Team Care: Increasing Access to Clinical Preventive Services in Los Angeles County
Source: Front Public Health. 2018 Feb 8;6:17. doi: 10.3389/fpubh.2018.00017 (PMC5809468; doi:10.3389/fpubh.2018.00017)
Supplement: Supplementary file 1 [file table_2.PDF]

Table A. Public opinions about team care – Internet panel survey, Los Angeles County, 2014

Sample Characteristics	Total Sample (n=1,044) Weighted Proportion[a]	Interested in team care (n=609) Weighted Proportion[a,b]	Interested in health navigator/coaches (n=446) Weighted Proportion[a,c]
**Gender**			
Male	0.49	0.48	0.49
Female	0.51	0.52	0.51
**Age**			
18-24	0.13	0.12	0.11
25-44	0.41	0.41	0.45
45-64	0.33	0.35	0.33
65+	0.14	0.12	0.11
**Race/ethnicity**			
Black/African American	0.09	0.08	0.09
White/Caucasian	0.31	0.30	0.28
Hispanic/Latino	0.43	0.47	0.48
Asian	0.16	0.14	0.15
Other	0.02	0.01	0.01
**Income** (annual before taxes)			
<$15,000	0.09	0.08	0.08
$15,000-$24,999	0.10	0.12	0.08
$25,000-$49,999	0.24	0.24	0.27
$50,000-$74,999	0.19	0.19	0.19
$75,000-$99,999	0.13	0.14	0.14
$100,000-$149,999	0.15	0.15	0.17
>$150,000	0.10	0.08	0.07
**Education**			
High school or less	0.29	0.27	0.27
Some college (no degree)	0.27	0.25	0.28
Technical school/2-year degree	0.10	0.12	0.11
Graduated 4-year college	0.22	0.23	0.22
Graduated with professional degree	0.12	0.13	0.11
Prefer not to answer	0.004	0.003	0.00
**Source of health insurance**			
Employer provided	0.48	0.49	0.46
Self-purchased	0.10	0.10	0.11
Medicare	0.12	0.10	0.10
Medi-CAL (Medicaid)	0.15	0.16	0.17
Military (e.g., Veterans Affairs)	0.01	0.002	0.004
Not insured at the time	0.11	0.12	0.14
Other/don’t know	0.03	0.03	0.03

**Purchased insurance through the Affordable Care Act exchange program**[d]			
Yes	0.44	0.45	0.38
No	0.56	0.55	0.62
**Type of health insurance**[e]			
Health Maintenance Organization (HMO)	0.49	0.48	0.52
Preferred Provider Organization (PPO)	0.42	0.42	0.39
Point of Service (POS)	0.02	0.02	0.01
Not sure/don't know	0.08	0.08	0.07

[a] Percent may not add up to 100% due to rounding.
[b] This question was only asked of respondents who reported that their healthcare provider does not operate team care (n=863). Analysis sample included those who responded – extremely interested, very interested, or somewhat interested (n=609).
[c] This question was only asked of respondents who reported not having a healthcare navigator or health coach (n=849). Analysis sample included those who responded – extremely interested, very interested, or somewhat interested (n=446).
[d] This question was only asked of respondents who self-purchased their health insurance (n=133 of total sample; n=86 of team care sample; n=68 of health navigator sample).
[e] This question was only asked of respondents who reported their source of health insurance was their employer or self-purchased (n=746 of total sample; n=443 of team care sample; n=315 of health navigator sample).

Technical Note: The Internet panel survey was conducted in June 2014 by Global Strategy Group, an online panel survey firm contracted by the Los Angeles County Public Health Department. Respondents were recruited by email through the vendor's panel of subscribers. Eligibility criteria included: (1) $\geq$ age 18 years, (2) live within the Los Angeles County jurisdiction, and (3) be willing to provide complete demographic information. Statistical weights were generated to account for various factors, including: (a) differential sampling rate for the subgroups (quota targets); (b) differential nonresponses; and (c) other factors such as region, education, marital status, adults and children in the household, body mass index, gender by age, and region by gender, age, race, and income.
